# Cerium oxide nanoparticles improve cotton salt tolerance by enabling better ability to maintain cytosolic K^+^/Na^+^ ratio

**DOI:** 10.1186/s12951-021-00892-7

**Published:** 2021-05-25

**Authors:** Jiahao Liu, Guangjing Li, Linlin Chen, Jiangjiang Gu, Honghong Wu, Zhaohu Li

**Affiliations:** 1 MOA Key Laboratory of Crop Ecophysiology and Farming System in the Middle Reaches of the Yangtze River, College of Plant Science & Technology, Huazhong Agricultural University, Wuhan, 430070 China; 2 College of Science, Huazhong Agricultural University, Wuhan, 430070 China; 3 College of Agronomy and Biotechnology, China Agricultural University, Beijing, 100083 China

**Keywords:** Nanoceria, Cotton, K^+^/na^+^ ratio, Photosynthesis, ROS, Salinity, Agriculture

## Abstract

**Background:**

Salinity is a worldwide factor limiting the agricultural production. Cotton is an important cash crop; however, its yield and product quality are negatively affected by soil salinity. Use of nanomaterials such as cerium oxide nanoparticles (nanoceria) to improve plant tolerance to stress conditions, e.g. salinity, is an emerged approach in agricultural production. Nevertheless, to date, our knowledge about the role of nanoceria in cotton salt response and the behind mechanisms is still rare.

**Results:**

We found that PNC (poly acrylic acid coated nanoceria) helped to improve cotton tolerance to salinity, showing better phenotypic performance, higher chlorophyll content (up to 68% increase) and biomass (up to 38% increase), and better photosynthetic performance such as carbon assimilation rate (up to 144% increase) in PNC treated cotton plants than the NNP (non-nanoparticle control) group. Under salinity stress, in consistent to the results of the enhanced activities of antioxidant enzymes, PNC treated cotton plants showed significant lower MDA (malondialdehyde, up to 44% decrease) content and reactive oxygen species (ROS) level such as hydrogen peroxide (H_2_O_2_, up to 79% decrease) than the NNP control group, both in the first and second true leaves. Further experiments showed that under salinity stress, PNC treated cotton plants had significant higher cytosolic K^+^ (up to 84% increase) and lower cytosolic Na^+^ (up to 77% decrease) fluorescent intensity in both the first and second true leaves than the NNP control group. This is further confirmed by the leaf ion content analysis, showed that PNC treated cotton plants maintained significant higher leaf K^+^ (up to 84% increase) and lower leaf Na^+^ content (up to 63% decrease), and thus the higher K^+^/Na^+^ ratio than the NNP control plants under salinity stress. Whereas no significant increase of mesophyll cell vacuolar Na^+^ intensity was observed in PNC treated plants than the NNP control under salinity stress, suggesting that the enhanced leaf K^+^ retention and leaf Na^+^ exclusion, but not leaf vacuolar Na^+^ sequestration are the main mechanisms behind PNC improved cotton salt tolerance. qPCR results showed that under salinity stress, the modulation of *HKT1* but not *SOS1* refers more to the PNC improved cotton leaf Na^+^ exclusion than the NNP control.

**Conclusions:**

PNC enhanced leaf K^+^ retention and Na^+^ exclusion, but not vacuolar Na^+^ sequestration to enable better maintained cytosolic K^+^/Na^+^ homeostasis and thus to improve cotton salt tolerance. Our results add more knowledge for better understanding the complexity of plant-nanoceria interaction in terms of nano-enabled plant stress tolerance.

**Graphic abstract:**

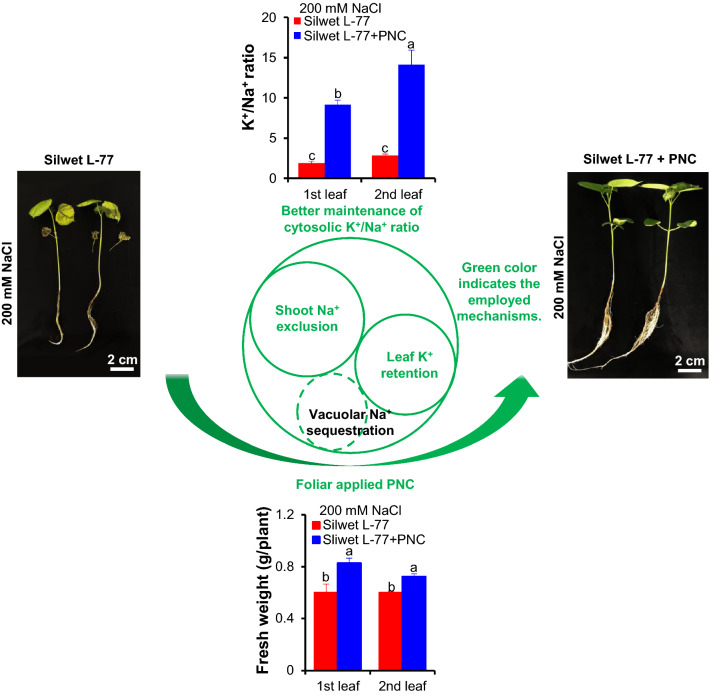

**Supplementary Information:**

The online version contains supplementary material available at 10.1186/s12951-021-00892-7.

## Background

Salinity is an environmental stress limiting agricultural production world-widely, causing the losses of billions of dollars per year [1]. Unlike drought and temperature stresses such as heat and cold which either happened in short period or can be addressed quickly through agricultural management practice, salinity is a long-lasting stress in the field. Salinity reduces crop yield, and decreases the quality of agricultural products e.g. seeds, fruits, and fibre [2–4]. Given the fact that breeding salt tolerant crop species is a time-consuming program and the practical approach such as irrigating saline soil with fresh water is not affordable in many areas, especially the semi-arid area, techniques which can help to improve crop salt tolerance in the production period could be an option for farming practice. Plant nanobiotechnology approach is an emerging technique to tune plant stress responses. Many nanomaterials have been reported to improve plant salt tolerance. For example, nanoparticles, i.e. CeO_2_, SeNP, TiO_2_, and AgNP improved salinity stress tolerance in plants such as barley [5], canola [6–8], potato [9], tomato [10, 11], broccoli [12] and *Arabidopsis* [13]. For example, Rossi et al. showed that compared with the non-nanoparticle control under salinity stress, 500 mg/Kg nanoceria application improves rapeseeds biomass by 45% [6]. Under salinity stress, applying multiwalled carbon nanotubes (MWCNTs) improves fresh weight of broccoli plants by about 70% compared with the control without MWCNTs treatment [12]. Thus, improving crop salt tolerance with nanomaterials could be a promising approach to enhance plant production in saline soils.

Under salinity stress, excess Na^+^ entered into the cell and caused massive K^+^ loss from the cell. Thus, the K^+^/Na^+^ ratio is a well-known hallmark for plant salt tolerance [14–16]. Also, the ability to maintain K^+^/Na^+^ ratio in plants under salinity is tightly associated with its salt tolerance. However, to date, previous work mainly focused on how nanoceria (cerium oxide nanoparticles) modulate either K^+^ retention [13] or Na^+^ detoxification [7]. Our previous work showed that nanoceria could improve plant salt tolerance via scavenging of over-accumulated ROS and modulating NSCC (non-selective cation channels) and KOR (K^+^ outward rectifying channels) activities to enable better mesophyll K^+^ retention [13]. Other researchers showed that nanoceria improved canola salt tolerance by enhancing plant photosynthetic performance and reducing the root apoplastic barrier to allow more Na^+^ being transferred into the shoot [7]. Nonetheless, it should be noticed that possible over-accumulation of Na^+^ in the shoot also could be an issue to plants since leaves are the main site for plant photosynthesis. Sequestrating Na^+^ from the cytosol to vacuole and using it for cheap osmoticum is an effective way to avoid the over-accumulation of Na^+^ in cell cytosol of plants under salinity stress [17, 18]. Another way to avoid shoot overaccumulation of cytosolic Na^+^ is to exclude it to the roots [19, 20]. Investigating the possible contribution of vacuolar Na^+^ sequestration and shoot Na^+^ exclusion in maintaining cytosolic K^+^/Na^+^ ratio and thus its role in nanoceria improved plant salt tolerance is the one of the aims of this work.

Cotton (*Gossypium hirsutum* L.) is an important cash and oil crop, and is also one of the most important fibre/textile crops. For example, in marketing year 2019, nearly 20 million bales of cotton which corresponds to ~ $7 billion were produced in the USA (https://www.ers.usda.gov/topics/crops/cotton-wool/cotton-sector-at-a-glance/) [21]. Although cotton is regarded as a moderate salt tolerant crop, it still suffers from salinity stress, especially in the semi-arid area. Salinity not only reduces cotton yield, but also impairs the quality of cotton fibre [22–24]. For example, cotton plants raised at high saline soil showed reduced fibre maturity such as linear density and maturity percent and ratio [25]. Compared with long term process of breeding salt tolerant species, improving cotton salt tolerance in its production period by plant nanobiotechnology approach could be an alternative option. Investigating whether plant nanobiotechnology approach could improve cotton plant salt tolerance is another aim to be addressed in this work.

Giving the fact that cotton is an important cash crop across the globe and salinity is a limiting factor affecting cotton yield and fibre quality, to our surprise, to date, only one paper tried to investigate the effect of cerium oxide nanoparticles on cotton salt tolerance through nanoceria seed treatment. An et al. found that nanoceria primed cotton seeds showed higher root length and biomass under salinity and associated it with the modulation of ROS and Ca^2+^ signaling pathways [26]. However, the experiments are conducted under paper roll condition. Besides the germination stage, the seedling stage is also crucial for cotton plants to survive under salinity stress. Nanoceria (cerium oxide nanoparticles) are potent ROS scavenger and are widely used in industry, medical research, and plant research [27–31]. Nanoceria have been reported to improve plant resistance/tolerance to various stress conditions, e.g. salinity [6, 7, 13], drought [32], light stress (such as highlight and UV [28]), and temperature stress (such as heat and chilling, [28]) etc. Whether nanoceria can help to improve salt tolerance in cotton plants and its associated mechanisms are worthy to be explored. Here, we hypothesized that nanoceria could help to scavenge over-accumulated ROS to maintain cytosolic K^+^/Na^+^ homeostasis through modulating Na^+^ exclusion, vacuolar Na^+^ sequestration or K^+^ retention, eventually improving cotton salt tolerance.

In this work, we investigated the biological responses such as phenotypic performance, chlorophyll content, photosynthetic performance, antioxidant enzyme activities, and leaf Na^+^ and K^+^ content in the first and second true leaves of cotton plants (two-leaf stage). Then, using confocal microscopy, the subcellular distribution of PNC, ROS level, subcellular Na^+^ and K^+^ level in leaf mesophyll cells in the first and second true leaves of cotton plants were studied. Also, qPCR experiments were performed to investigate the relative expression level of genes related to Na^+^ and K^+^ transport.

## Materials and methods

### Plant growth

Cotton plants (variety Xinluzao 74, XLZ 74) at the two-leaf stage (two true leaves were fully expanded) were grown in Hoagland solution. Seeds were sown in pots (10 × 10 cm) filled with standard soil mix (Xingyuxing, Wuhan, China). After the unfolding of the cotyledons, plants with similar size were transplanted into a tray filled with Hoagland solution. Plants were grown in growth room with the settings: 200 μmol m^−2^ s^−1^ photosynthetic active radiation (PAR), 28 ± 1 °C (day time) and 25 ± 1 °C (night time), 70% relative humidity, and 14/10 h as the day/night regime. Hoagland solution was refreshed once every five days. After cotton plants reached the two-leaf stage, six plants with similar size were selected and transplanted in a tray with 5 L Hoagland solution before treatment. For salinity stress experiments, 200 mM NaCl solution was applied (mimicking the salt level in the salinized field in Xingjiang province which is the main cotton production area in China) to treat the cotton plants (foliar delivered with or without PNC) for another five days. Moreover, to test the biocompatibility of PNC, cotton plants treated with PNC + Silwet L-77 or Silwet L-77 alone were grown under non-saline condition for 28 days.

### Synthesis and characterization of PNC

The synthesis and characterization of poly (acrylic acid) coated cerium oxide nanoparticles (PNC) were followed the method described in our previous publications [33, 34]. Briefly, 4.5 g poly (acrylic acid) and 1.08 g cerium(III) nitrate were respectively dissolved in 5.0 mL and 2.5 mL deionized water. The two solutions were mixed thoroughly at 2, 500 rpm for 15 min using a vortex mixer. The resulted mixture was then added dropwise to 15 mL ammonium hydroxide solution (30%) in a 50 mL beaker, and the reaction was kept by stirring at 500 rpm for 24 h at 25 °C. Then, the solution was centrifuged at 4000 rpm for 1 h to remove any debris and large agglomerates. The supernatant was collected by using 10 K Amicon cells (4, 500 rpm, six cycles) to purify the free polymers and other reagents, and was denoted as PNC solution. The final PNC solution was stored in a refrigerator (4 °C) for up to two weeks. The absorbance of final PNC solution at 271 nm was measured by the UV–VIS spectrophotometer, and the concentration was calculated using Beer-Lambert’s law A = εCL. A, the PNC absorbance at 271 nm; ɛ, the molar absorption coefficient of PNC (3 cm^−1^ mM^−1^) [35, 36]; L, the optical path length (cuvette width, 1 cm in this method); and C is the molar concentration of the measured PNC. All chemicals are from Sigma Aldrich, unless otherwise specified.

The hydrodynamic diameter (DLS size) and zeta potential were determined by 90 Plus PALS (Brookhaven Instruments Corporation, USA). 20 μL of PNC (0.45 mM) was mounted on a holey carbon-coated copper grid, and then the PNC TEM imaging was done by a FEI Talos microscope operating at 300 kV.

### DiI labeling of PNC

The labelling of PNC with DiI (1,1ʹ-dioctadecyl-3,3,3ʹ,3ʹ-tetramethylindocarbocyanine) was followed the method described in our previous publications [33, 34]. Briefly, in a 20 mL glass vial, 4 mL, 0.5 mM PNC and 200 μL, 0.3 mg/mL DiI (in DMSO) was mixed at 1000 rpm for 1 min. The resulting mixture was purified using 10 kDa filter (4, 500 rpm for 5 min at least five times) to remove the free chemicals. The final solution was labelled as DiI-PNC and was stored in a refrigerator at 4 °C for further use.

### Foliar delivery of PNC and DiI-PNC to cotton plant

Foliar delivery of PNC and DiI-PNC to cotton leaves was followed the method of our previous publication [35] with minor modifications. Briefly, PNC and DiI-PNC formulation were complexed with the surfactant Silwet L-77 (0.05%, Yuanye, Shanghai, China). 0.1 mL, 0.9 mM PNC and DiI-PNC were foliar delivered to each leaf by using a 1000 μL pipette. The first and second true leaves (the order of expansion) of cotton plants were foliar delivered with Silwet L-77 (also denoted as non-nanoparticle control, NNP), PNC + Silwet L-77 and DiI-PNC + Silwet L-77. During the foliar delivery of PNC, press the pipette but hold the prepared solution, and then gently move it around the whole leaf to ensure the homogenous delivery of the prepared solution. The 1,000 μL pipette tips was cut about 0.3 cm from the top to remove the sharp tip to avoid the possible physical damage during the foliar delivering of the prepared solution. After the foliar delivery of the prepared solution, the excess solution on the leaf were removed immediately. To avoid confounding effects during photosynthesis, after the foliar delivery of the prepared solution, cotton leaves were incubated in the room light for 3 h to allow the incubation and plant adaption. After 3 h incubation, the plants were put back to the growth chamber with 200 μmol m^−2^ s^−1^ PAR lights and then the 200 mM NaCl was applied.

### Laser confocal microscopy imaging

To quantify ROS in vivo, leaf discs (diameter, 5 mm) from the first and second true leaves of the stressed plants (200 mM NaCl, 5 days) were incubated with 25 μM 2′,7′-dichlorodihydrofluorescein diacetate (H_2_DCFDA, staining of H_2_O_2_) or 10 μM dihydroethidium (DHE, staining of ^⋅^O_2_^−^) dyes in 1.5 mL tubes for 30 min under darkness. Confocal imaging was performed as described in our previous publication [33] with modifications. After the above-mentioned 30 min incubation, the leaf discs were mounted on microscope slides with mounting medium and sealed with a coverslip. Leaf discs were imaged by using Leica SP8 spectral confocal laser scanning microscope. Three plants/biological replicates (2 leaf discs for each plant) were used. The imaging settings were as follows: 488 nm laser excitation; PMT1, 500–600 nm (for DCF and DHE fluorescence); PMT2, 700–785 nm (for chloroplast fluorescence). Imaging of Na^+^ and K^+^ distribution in the cytosol and vacuole was performed using 20 μM Na^+^ dye CoroNa Green AM (Thermo Fisher Scientific), 20 μM K^+^ dye APG-2 AM (asante potassium green-2AM, Abcam Biotechnology company), and 20 μM membrane dye FM 4–64 {*N*-(3-triethykammoniumpropyl)-4Ĳ6-[4-(diethylamino)phenyl)hexatrienyl] pyridinium dibromide, Thermo Fisher Scientific} following our previous publications [13, 37]. The CoroNa Green AM and APG-2 AM fluorescent dyes were dissolved in pure DMSO, and then respectively diluted with buffer solution (10 mM KCl, 5 mM Ca^2+^-MES, pH 6.1) and TES {2-[(2-Hydroxy-1,1-bis(hydroxymethyl)ethyl)amino]ethanesulfonic acid; 10 mM, pH 7.5} to working concentration. As described above, cotton leaf discs were prepared and incubated with CoroNa Green AM + FM4-64 or APG-2 AM + FM-4–64 in 1.5 mL Eppendorf tubes for 2 h and 2.5 h in darkness, respectively. After incubation, leaf discs were rinsed in ddH_2_O and mounted on microscope slides for confocal imaging. The settings of Na^+^ and K^+^ confocal imaging were: 488 nm laser excitation; PMT1: 500–550 nm (for Na^+^ confocal imaging) or 520–560 nm (for K^+^ confocal imaging); PMT2: 610–630 nm for (membrane imaging). Obtained confocal images were analysed with Fiji.

The visualization of DiI-PNC in cotton leaves was also performed by using Leica SP8 spectral confocal laser scanning microscope. Briefly, after 3 h incubation of the foliar delivered DiI-PNC with the leaves of the cotton plant, leaf discs (diameter, 5 mm) from the first and second true leaves were made and mounted on the glass slides. PFD (perfluorodecalin) were used to enable better imaging quality. After sealing the slides with a coverslip, the samples are ready for confocal imaging. The imaging settings for DiI-PNC visualization in cotton leaves were as follows: 514 nm laser excitation; PMT1, 550–615 nm (for DiI-PNC fluorescence); PMT2, 700–750 nm (for chloroplast fluorescence). Colocalization between DiI-PNC and chloroplasts was analysed with LAS AF Lite software follow the method described in our previous publications [33]. Three lines of sections were drawn across the ROI (region of interest) with 40 μm interval on the confocal images. The colocalization rate between PNC and chloroplasts was recorded by calculating the proportion of DiI-PNC fluorescence which are overlapped with chloroplast fluorescence emission peaks out of all chloroplast peaks.

### Cotton plant performance under salinity stress

The chlorophyll content index (CCI) of the first and second true leaves was daily monitored in salt stressed (200 mM NaCl) cotton plant with or without PNC. CCI measurements were performed using a chlorophyll meter (SPAD-502 PLUS, Konica Minolta, Japan) with each leaf being measured at three different points (each data point was composed of at least three CCI readouts). Plant height was also daily measured for 5 days after the onset of salinity stress. Plant height is defined as the distance between the growth point of the top leaf and the ground/the black foam board on top of the tray [38]. Biomass was determined after 5 days salinity stress. Plants were drying firstly at 105 °C for 30 min, and then at 85 °C for 72 h until reaching the constant weight. The phenotype images of the salt stressed cotton plants with and without PNC were taken by a Nikon D810 camera.

### Determination of hydrogen peroxide (H_2_O_2_), superoxide anion (O_2_^⋅−^) and malondialdehyde (MDA) content

Measurement of H_2_O_2_ content in leaf sample was done following a widely accepted method [39] with minor modifications. Approximately 200 mg of fresh samples were placed in liquid nitrogen and then ground with 2 mL cold acetone. The mixture was then centrifuged (3000 rpm) for 15 min. 1 mL of Ti(SO_4_)_2_ (5%/W/V in concentrated HCl) was added to the supernatant. After shaking, the samples were then centrifuged (3000 rpm) and the precipitates were solubilized in 1 mL H_2_SO_4_. The absorbance of the final solutions was measured at 415 nm vis UV–Vis spectrophotometer.

Estimation of superoxide anion radicals (O_2_^−^) was done following a widely accepted method [40] with minor modifications. NH_2_OH was used as a probe for O_2_^−^, being oxidized to NO_2_^−^. NO_2_^−^ can react with α-naphthylamine and sulfamic acid to turn the mixture to red colour. Then, the absorbance of the mixture can be measured at 530 nm by UV–Vis spectrophotometer. The assessment of MDA content was done by following the method described in previous publication [41]. Briefly, 200 mg fresh samples were homogenized with 5 mL TCA having 0.25% 2-thiobarbituric acid (TBA). After incubating at 90 °C for 30 min, the mixture was immediately cooled down, and then was centrifugated at 8000*g* for 15 min. The absorbance of the solution was measured at 450 nm, 532 nm and 600 nm.

### Measurement of antioxidant enzymes activities

To determine the activity of SOD, CAT and POD, the first and second true leaves of the salt stressed cotton plants (200 mM NaCl, 5 days) were separately collected. For the determination of SOD activity, the freshly collected leaf samples were ground with PBS buffer (pH 7.8). The supernatant was collected following the centrifugation at 12,000*g* for 20 min. The supernatant was mixed with EDTA-Riboflavin, NBT and methionine. The mixture was incubated under 200 μmol m^−2^ s^−1^ lx light for 20 min. The absorbance of the final mixture was measured at 560 nm by a UV–Vis spectrophotometer (UV 1800PC, AOE, Shanghai, China). SOD enzyme activity was calculated by using the measured absorbance value following Eq. 1:

1 $$ {\text{SOD activity}} = \left( {{\text{A}}_{{\text{b}}} - {\text{A}}_{{\text{s}}} } \right) \times {\text{V}}_{{\text{T}}} /({\text{A}}_{{\text{b}}} \times 0.{5} \times {\text{W}}_{{\text{s}}} \times {\text{V}}_{{\text{s}}} ). $$

where A_b_ and A_s_ are the blank control and samples’ absorbance value, respectively. V_T_ is the volume of crude leaf extraction mixture. W_s_ is fresh weight and V_s_ is the used volume during the sample measurement.

For the determination of POD activity, the collected samples were ground with PBS buffer (pH 5.5). The supernatant was collected following the centrifugation at 3000 rpm for 10 min. For POD activity measurement, the solution was prepared with mixing the supernatant of the crude leaf extraction mixture, 20% TCA, 50 mM guaiacol solution, PBS buffer (pH 5.5, 50 mM), and 2% H_2_O_2_. The POD activity was measured by calculating the averaged decrease of the recorded absorbance value at 470 nm (1 record/1 min, 3 min) by a UV–Vis spectrophotometer (UV 1800PC, AOE, Shanghai, China). Calculations were done by following previous methods [42], using the absorbance coefficient of 26.6 mM^−1^ cm^−1^.

For CAT activity measurement, the collected samples were ground with PBS buffer (pH 7.8). The supernatant was collected following the centrifugation at 4000 rpm for 15 min. The supernatant of the crude leaf extraction mixture was vortexed with PBS buffer (pH 7.8) and 10 mM H_2_O_2_. The CAT enzyme activity was calculated based on the averaged decrease of the recorded absorbance value at 240 nm (1 record/1 min, 4 min) by a UV–Vis spectrophotometer (UV 1800PC, AOE, Shanghai, China). Calculations were done by following previous methods [43], using the absorbance coefficient of 43.6 M^−1^ cm^−1^.

### Measurement of chlorophyll content and photosynthetic parameters

Leaf samples (the first and second true leaves) was mixed with a solution containing acetone and ethanol (1:1) for 24 h at dark condition on a shaker (50 rpm). Following centrifugation (2000 rpm, 10 min), the supernatant was collected. By using a UV–Vis spectrophotometer (UV 1800PC, AOE, Shanghai, China), the absorbance of the supernatant was measured at 644 nm and 662 nm to determine the content of chlorophyll a and chlorophyll b. The chlorophyll a and b content were calculated using the following equations:

2 $$ {\text{Chlorophyll a content }} = { 9}.{784} \times {\text{A}}_{{{662}}} {-}0.{99} \times {\text{A}}_{{{644}}} . $$

3 $$ {\text{Chlorophyll b content}} = {21}.{426} \times {\text{A}}_{{{644}}} {-}{4}.{65} \times {\text{A}}_{{{662}}} . $$

where A_662_ and A_644_ are the absorbance value measured at 662 nm and 644 nm, respectively.

Photosynthesis rate, intercellular CO_2_ concentration, stomatal conductivity, and transpiration rate of the first and second true leaves were measured by using a portable photosynthetic apparatus Li-6400 XT at D0 (200 mM NaCl, day 0) and D5 (200 mM NaCl, day 5). D0 here denotes that the photosynthetic performance data are collected from plants without salt stress (the salt stress was not yet onset). After the measurement of photosynthetic performance at D0, plants were foliar applied with PNC + Silwet L-77 solution or the Silwet L-77 solution alone. After 3 h incubation, 200 mM NaCl were then onset. Data measured in plants after 5 days salt stress (200 mM NaCl) were then denoted as D5. The measurement settings are: 1500 μmol m^−2^ s^−1^ photosynthetic photon flux density, 400 μmol mol^−1^ CO_2_ concentration, and 25 °C leaf temperature.

### Estimation of K^+^ and Na^+^ content

For the estimation of leaf K^+^ and Na^+^ content, samples of the first and second true leaves were milled with a grinder and filtered through a 0.5 mm sieve. 0.2 g of ground samples were then digested for 1.5 h in concentrated H_2_SO_4_ (18.4 M). After cooling down, 30% H_2_O_2_ was added into the digested samples to get the transparent mixture solution. The mixture was digested for another 1 h to make sure the H_2_O_2_ decompose completely. Flame photometer (FP6431, Jiangke, Shanghai, China) was used to determine the content of K^+^ and Na^+^ in the samples. The setup of standard curve can be found in the literatures elsewhere.

### qPCR

Total RNA was isolated using the RNAprep Pure Plant Kit (DP441, Tiangen, Beijing, China). 2 μg of total RNA was reversely transcribed into cDNA using the TRUEscript first Strand cDNA Synthesis Kit (PC5402, Aidlab, Beijing, China). The amplification of qRT-PCR products was performed in a reaction mixture of 12.5 μL SYBR Green qPCR Mix (PC3302, Aidlab, Beijing, China) according to the manufacturer’s instructions. The qRT-PCR analysis was performed on the Bio-Rad CFX Connect Real-Time PCR System (Bio-Rad, California, USA). Three biological replicates and three technical replicates was used for each investigated gene. The relative gene expression was calculated using the 2^−ΔΔCt^ method. The primers used for qRT-PCR are shown in Additional file 1: Table S1 [44–46].

### Statistical analysis

All data were represented as mean ± SE and were analysed using SPSS 23.0. Comparisons were performed by either one-way ANOVA based on Duncan’s multiple range test (two tailed) or independent samples t-test (two tailed). * for *P* < 0.05, ** for *P* < 0.01, and *** for *P* < 0.001. Different lowercase letters mean the significance at *P* < 0.05.

## Results

### PNC characterization and its distribution in cotton leaves

**Fig. 1:**
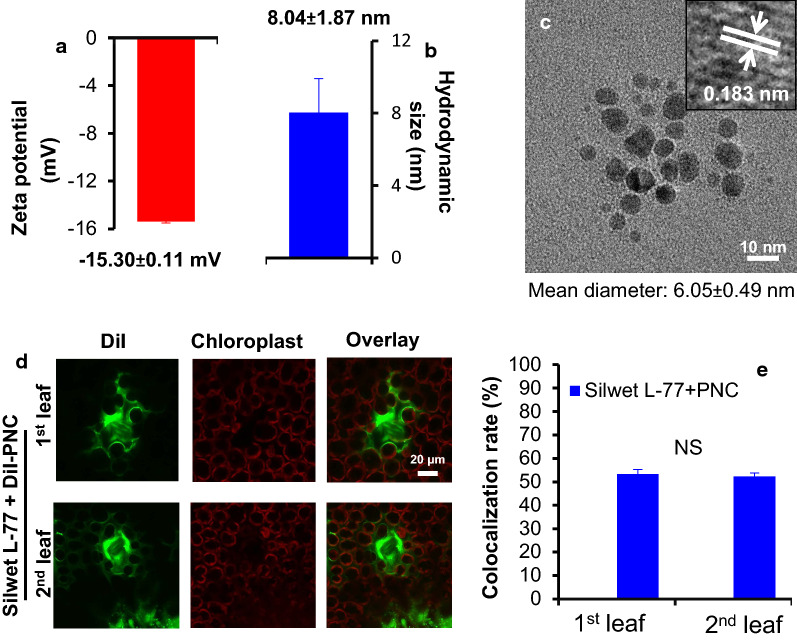
PNC characterization and its colocalization with chloroplasts. **a**, **b** The size and charge of the synthesized PNC. Mean ± SE (n = 3). **c** The TEM imaging of the synthesized PNC. **d** Confocal imaging results showing the distribution of PNC in the first and second true leaves of the cotton plants. **e** The calculated colocalization rate between PNC and chloroplasts in the first and second true leaves of the cotton plants. Mean ± SE (n = 3). Different lower-case letters indicate the significance level at 0.05. *NS* no significant difference

Hydrodynamic diameter measurements showed that the size of PNC is 8.04 ± 1.87 nm (Fig. 1a). TEM imaging results showed that the core of the synthesized PNC is spherical with an average diameter of 6.05 ± 0.49 nm (Fig. 1b). The zeta potential measurement showed that the surface charge of the synthesized PNC is − 15.30 ± 0.11 mV (Fig. 1c). The synthesized PNC have a peak absorbance at 271 nm (Additional file 1: Figure S1a). Besides the peak absorbance at 271 nm, DiI-PNC have another two peaks at 557 and 517 nm, indicating the successful conjugation of DiI to PNC (Additional file 1: Figure S1a). The red colour of DiI-PNC further confirmed the successful synthesis of DiI-PNC (Additional file 1: Figure S1b). Confocal imaging results showed that the colocalization rate between DiI-PNC and chloroplasts is 53.34 ± 1.90% and 52.32 ± 1.51% in the first and second true leaves, respectively (Fig. 1d, e). No DiI fluorescence signal was detected in cotton leaves treated with Silwet-L77 (Additional file 1: Figure S2).

### PNC improved the performance of cotton under salinity stress

**Fig. 2:**
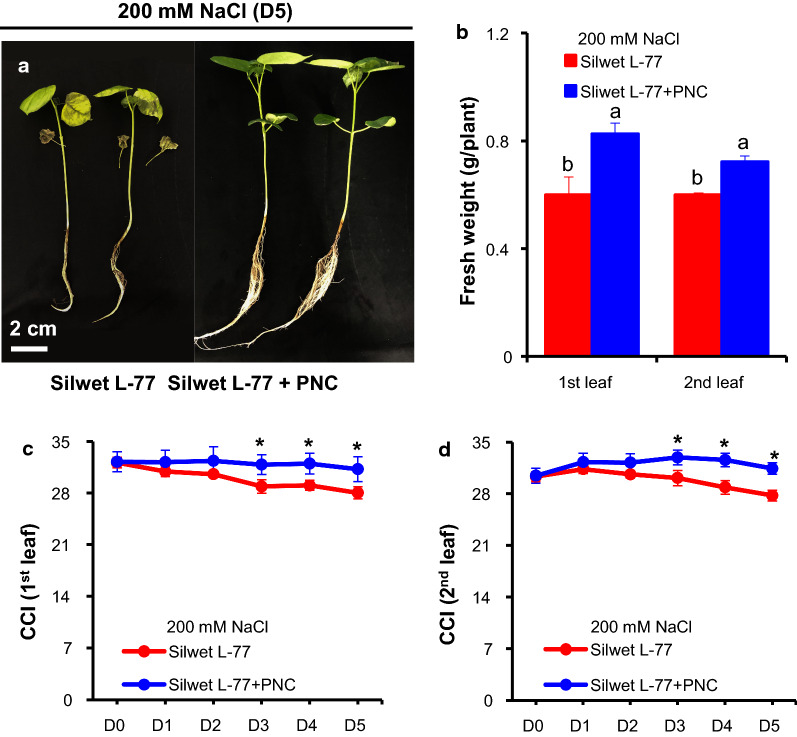
The phenotypic performance and chlorophyll content of salt stressed (200 mM NaCl, 5 days) cotton plants with and without PNC treatment. **a** Phenotypic performance of salt stressed cotton plants with and without PNC treatment. **b** The fresh weight of salt stressed cotton plants with and without PNC treatment. **c**, **d** CCI (chlorophyll content index) readout in the first (**c**) and second (**d**) true leaves of salt stressed cotton plants with and without PNC treatment. Mean ± SE (n = 6). Different lower-case letters indicate the significance level at 0.05. *Means *P* < 0.05. The comparison was made between PNC treated cotton plants and the NNP control at each day

Compared to cotton plants without PNC treatment, under 200 mM NaCl, the plants with foliar delivered PNC showed obvious better phenotypic performance (Fig. 2a, Additional file 1: Figure S3a–c) and significant higher biomass such as leaf fresh weight (Fig. 2b), dry weight (Additional file 1: Figure S3d) and whole plant weight (Additional file 1: Figure S3e), either in the first or the second true leaf. Under 200 mM NaCl, compared with the 0.60 ± 0.09 and 0.60 ± 0.01 g/plant in the NNP control group, the fresh weight of the first and the second true leaves in PNC treated plants is 0.83 ± 0.07 and 0.72 ± 0.04 g/plant, respectively. Also, under 200 mM NaCl, PNC treated cotton plants showed significant higher chlorophyll content (indicated by CCI, chlorophyll content index) in both the first and the second true leaves than the NNP group (Silwet L-77 treated plants) (Fig. 2c, d). The results of chlorophyll a (0.60 ± 0.03 and 0.91 ± 0.04 mg/g in the first true leaf of salt stressed plants with and without PNC, 0.70 ± 0.03 and 1.17 ± 0.05 mg/g in the second true leaf of salt stressed plants with and without PNC) and chlorophyll b (0.21 ± 0.01 and 0.32 ± 0.02 mg/g in the first true leaf of salt stressed plants with and without PNC, 0.25 ± 0.01 and 0.42 ± 0.02 mg/g in the second true leaf of salt stressed plants with and without PNC) content (Additional file 1: Figure S4a, b) are also consistent with the CCI results. Under no saline conditions, no significant difference of the phenotypic performance and chlorophyll content (indicated by chlorophyll content index) were observed in cotton plants treated with and without PNC (Additional file 1: Figure S5a–c).

### PNC improved the photosynthetic performance of cotton under salinity stress

**Fig. 3:**
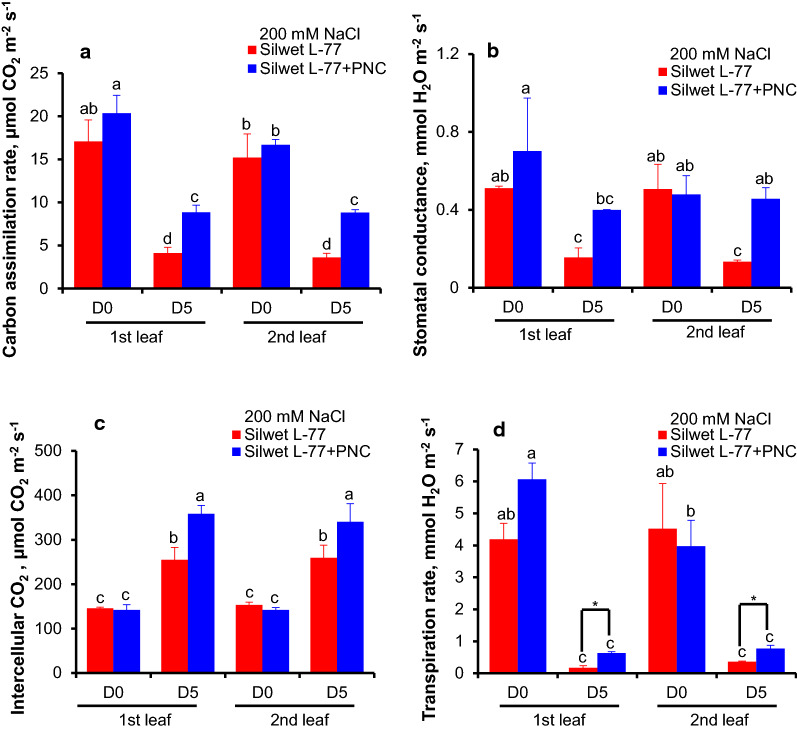
The photosynthetic performance of cotton plants with and without PNC treatment, before and after salt stress (200 mM NaCl, 5 days). **a**–**d** Carbon assimilation rate (**a**), stomatal conductance (**b**), intercellular CO_2_ (**c**), and transpiration rate (**d**) of the first and second true leaves of cotton plants with and without PNC treatment before and after salt stress. Mean ± SE (n = 8). Different lower-case letters indicate the significance level at 0.05

After five days salinity stress (D5, 200 mM NaCl), PNC treated cotton plants showed significant higher carbon assimilation rate in the first true leaf (8.84 ± 0.82 vs 4.10 ± 0.69 μmol CO_2_ m^−2^ s^−1^) and the second true leaf (8.83 ± 0.32 vs 3.62 ± 0.48 μmol CO_2_ m^−2^ s^−1^) than the control group (Fig. 3a). Similarly, compared with the control group under 200 mM NaCl, 5 days, PNC treated cotton plants showed significant higher stomatal conductance in the first true leaf (0.40 ± 0.01 vs 0.16 ± 0.05 mmol H_2_O m^−2^ s^−1^) and the second true leaf (0.46 ± 0.06 vs 0.13 ± 0.01 mmol H_2_O m^−2^ s^−1^) (Fig. 3b). Figure 3c showed that after five days salt stress, the PNC treated plants have significant higher intercellular CO_2_ than the control group, in both the first (358.3 ± 19.20 vs 255.0 ± 27.68 μmol CO_2_ m^−2^ s^−1^) and the second (340.67 ± 40.56 vs 259.67 ± 27.97 μmol CO_2_ m^−2^ s^−1^) true leaves. Similarly, after salinity stress, the transpiration rate of the PNC treated plants is significantly higher than the control group, in both the first (0.63 ± 0.43 vs 0.17 ± 0.07 μmol CO_2_ m^−2^ s^−1^) and the second (0.77 ± 0.10 vs 0.36 ± 0.02 μmol CO_2_ m^−2^ s^−1^) true leaves (Fig. 3d). At day 0 (D0, salinity stress is not yet onset), no significant difference of carbon assimilation rate, stomatal conductance, intercellular CO_2_ and transpiration rate was found between PNC treated cotton plants and the NNP control group, either in the first or the second true leaf (Fig. 3a–d).

### PNC improved the ROS scavenging ability of cotton under salinity stress

**Fig. 4:**
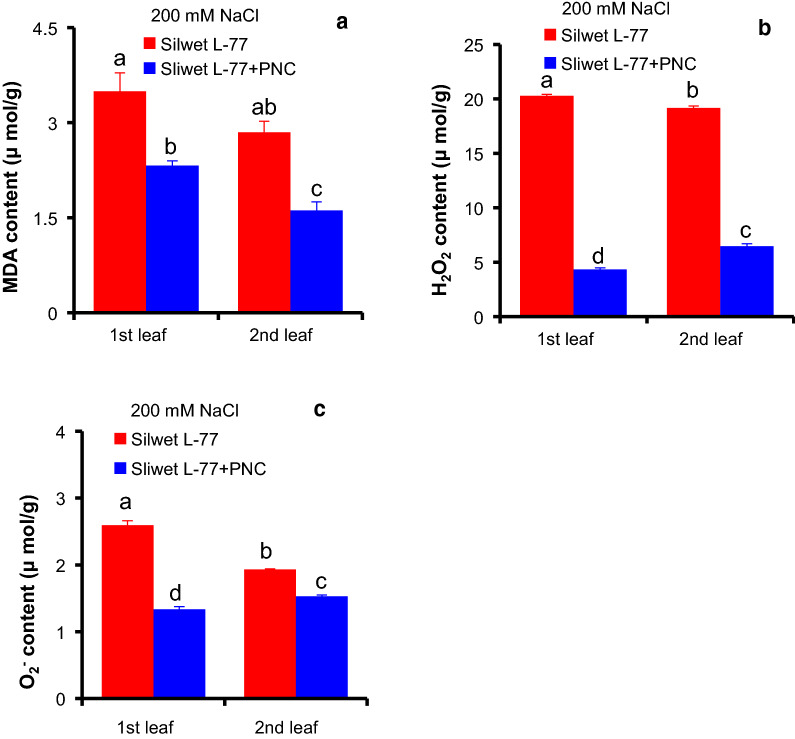
The MDA and ROS content in salt stressed (200 mM NaCl, 5 days) cotton plants with and without PNC treatment. **a**–**c** malondialdehyde (MDA) content (**a**), H_2_O_2_ content (**b**), and O_2_^−^ content (**c**) of the first and second true leaves of salt stressed cotton plants with and without PNC treatment. Mean ± SE (n = 12). Different lower-case letters indicate the significance level at 0.05

**Fig. 5:**
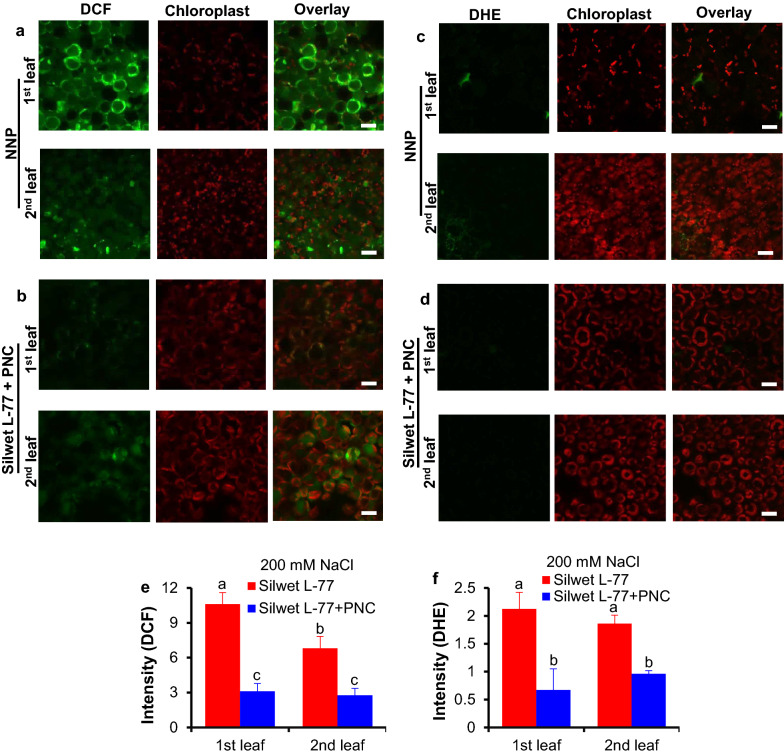
Confocal imaging of ROS fluorescent dye intensity in salt stressed (200 mM NaCl, 5 days) cotton plants with and without PNC treatment. a and b, DCF (largely indicating H_2_O_2_) fluorescent dye intensity of the first and second true leaves of salt stressed cotton plants without (**a**) and with (**b**) PNC treatment. **c**, **d** DHE (indicating ^⋅^O_2_^−^) fluorescent dye intensity of the first and second true leaves of salt stressed cotton plants without (**c**) and with (**d**) PNC treatment. e and f, the quantified DCF (**e**) and DHE (**f**) fluorescence intensity of the first and second true leaves of salt stressed cotton plants with and without PNC treatment. Mean ± SE (n = 3). Different lower-case letters indicate the significance level at 0.05. Scale bar is 20 μm. *NNP* non-nanoparticle control

After 5 days’ salt stress (200 mM NaCl), compared with the NNP control group, PNC treated cotton plants showed significant lower content of malondialdehyde (MDA), hydrogen peroxide (H_2_O_2_) and superoxide anion (^⋅^O_2_^−^) in either the first (3.49 ± 0.29 vs 2.32 ± 0.07 μmol/g for MDA, 20.30 ± 0.12 vs 4.34 ± 0.14 μmol/g for H_2_O_2_, 2.60 ± 0.07 vs 1.34 ± 0.04 μmol/g for ^⋅^O_2_^−^) or the second (2.85 ± 0.17 vs 1.61 ± 0.14 μmol/g for MDA, 19.18 ± 0.17 vs 6.47 ± 0.23 μmol/g for H_2_O_2_, 1.93 ± 0.01 vs 1.53 ± 0.02 μmol/g for ^⋅^O_2_^−^) true leaf (Fig. 4a–c). Confocal imaging results further confirmed the results. Using DCFDA (indicating H_2_O_2_) and DHE (indicating ^⋅^O_2_^−^) fluorescent dye, Fig. 5 showed that compared with the NNP control group, PNC helped to scavenge more ROS in both the first and the second true leaves of salt stressed cotton plants. It shows that PNC treated cotton plants have significant lower DCF and DHE fluorescent dye intensity in either the first (10.61 ± 1.05 vs 3.11 ± 0.68 and 2.12 ± 0.30 vs 0.67 ± 0.38 for H_2_O_2_ and ^⋅^O_2_^−^, respectively) or the second (6.82 ± 0.69 vs 2.77 ± 0.59 and 1.86 ± 0.15 vs 0.96 ± 0.05 for H_2_O_2_ and ^⋅^O_2_^−^, respectively) true leaf than the control group after the treatment of 200 mM NaCl, 5 days (Fig. 5a–c).In contrast to leaf MDA and ROS content, after 5 days’ salt stress (200 mM NaCl), the activities of SOD (superoxide dismutase), CAT (catalase) and POD (peroxidase) in the first (227.38 ± 9.89 vs 343.81 ± 16.95 U/g for SOD, 0.76 ± 0.03 vs 1.34 ± 0.04 mmol H_2_O_2_/mg protein/min for CAT, 4.30 ± 0.06 vs 6.85 ± 0.05 μmol tetra-gualacol /mg protein /min for POD) and the second (252.34 ± 7.08 vs 386.16 ± 3.15 U/g for SOD, 0.95 ± 0.04 vs 2.04 ± 0.09 mmol H_2_O_2_/mg protein/min for CAT, 6.28 ± 0.10 vs 7.21 ± 0.06 μmol tetra-gualacol /mg protein /min for POD) true leaves are significantly higher in PNC treated cotton plants than the control group (Additional file 1: Figure S6a–c).

### PNC helped to maintain K^+^/Na^+^ ratio in cotton under salinity stress

**Fig. 6:**
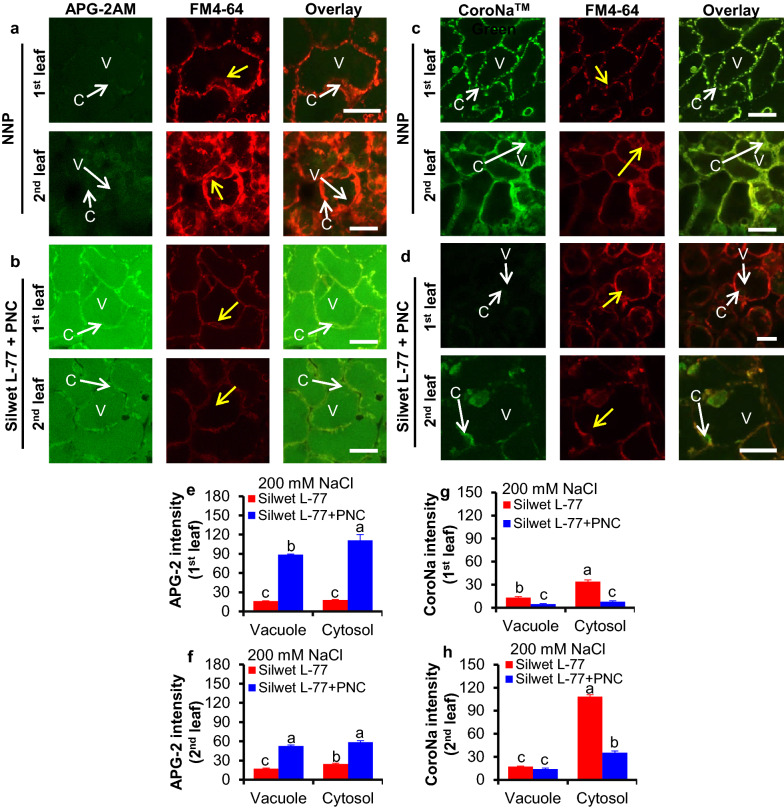
Confocal imaging of K^+^ and Na^+^ fluorescent dye intensity in salt stressed (200 mM NaCl, 5 days) cotton plants with and without PNC treatment. a and b, APG-2AM (indicating K^+^) fluorescent dye intensity of the first and second true leaves of salt stressed cotton plants without (**a**) and with (**b**) PNC treatment. c and d, CoroNa™ (indicating Na^+^) fluorescent dye intensity of the first and second true leaves of salt stressed cotton plants without (**c**) and with (**d**) PNC treatment. e and f, the quantified vacuolar and cytosolic APG-2AM fluorescent dye of the first (**e**) and second (**f**) true leaves of salt stressed cotton plants with and without PNC treatment. **g**, **h** The quantified vacuolar and cytosolic CoroNa™ fluorescent dye of the first (**g**) and second (**h**) true leaves of salt stressed cotton plants with and without PNC treatment. Mean ± SE (n = 3). Different lower-case letters indicate the significance level at 0.05. C means the cytosol, M means vacuole membrane, and V means the vacuole. The yellow arrows point to the tonoplast membrane. Scale bar is 20 μm. *NNP* non-nanoparticle control

**Fig. 7:**
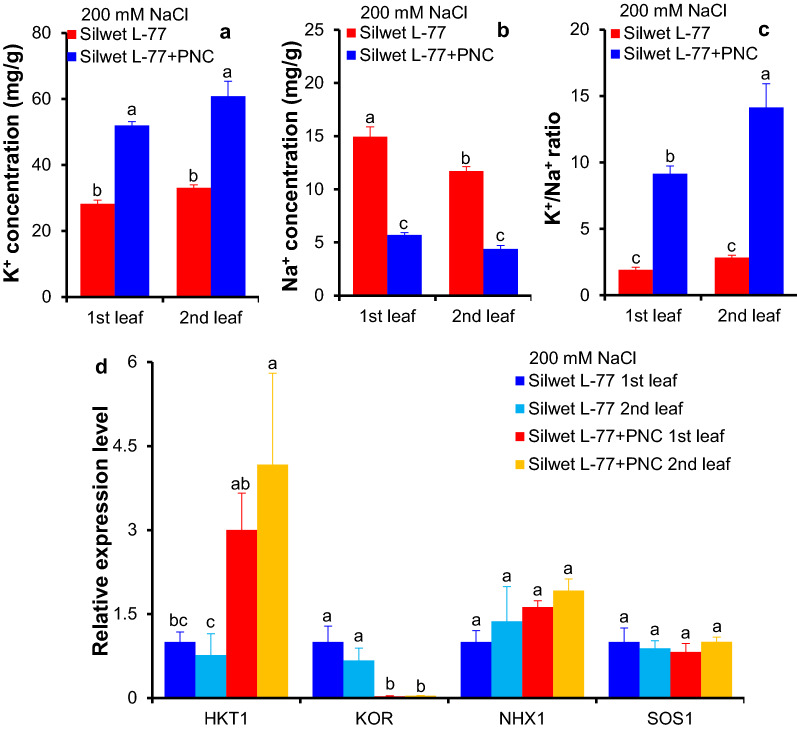
Leaf K^+^ and Na^+^ content, K^+^/Na^+^ ratio and the relative expression of ion channel/transporter genes related to K^+^ and Na^+^ transport in salt stressed (200 mM NaCl, 5 days) cotton plants with and without PNC treatment. **a**–**c** K^+^ content (**a**), Na^+^ (**b**) content and K^+^/Na^+^ ratio (**c**) in the first and second true leaves of salt stressed cotton plants with and without PNC treatment. Mean ± SE (n = 12). **d** The relative expression of ion channel/transporter genes related to K^+^ and Na^+^ transport in the first and second true leaves of cotton plants with and without PNC treatment, before and after salt stress. Mean ± SE (n = 3). Different lower-case letters indicate the significance level at 0.05

After 200 mM NaCl treatment for five days, PNC treated cotton plants showed significant higher APG-2 (K^+^ fluorescent dye) intensity in the cytosol (111.11 ± 8.92 vs 17.98 ± 0.77 and 58.57 ± 2.41 vs 24.61 ± 1.10 for the first and the second true leaves, respectively) and the vacuole (88.20 ± 0.95 vs 16.20 ± 0.53 and 52.66 ± 1.65 vs 17.50 ± 0.78 for the first and the second true leaves, respectively) of the mesophyll cells than the NNP control group (Fig. 6a, b, e, f). This is in accordance with the results of total leaf K^+^ content, showing that under salinity stress (200 mM NaCl, five days), PNC treated cotton plants have significant higher leaf K^+^ content in both the first (51.99 ± 1.19 vs 28.22 ± 1.18 mg/g) and the second (60.85 ± 4.58 vs 33.14 ± 0.87 mg/g) true leaves than the NNP control group (Fig. 7a). Whereas, after five days’ salt stress, CoroNa Green (Na^+^ fluorescent dye) intensity in the cytosol (33.89 ± 2.24 vs 7.93 ± 1.10 and 108.45 ± 2.48 vs 35.54 ± 2.11 for the first and the second true leaves, respectively) and the vacuole (13.24 ± 1.28 vs 4.62 ± 0.85 and 17.43 ± 0.73 vs 14.23 ± 1.32 for the first and the second true leaves, respectively) of the mesophyll cells is lower in the PNC treated cotton plants than the NNP control group (Fig. 6c, d, g, h). This is in consistent with the results of total leaf Na^+^ content, showing that under salinity stress (200 mM NaCl, five days), leaf Na^+^ content in both the first (14.95 ± 0.91 vs 5.71 ± 0.22 mg/g) and the second (11.70 ± 0.42 vs 4.38 ± 0.32 mg/g) true leaves is significant lower in PNC treated cotton plants than the NNP control group (Fig. 7b). No significant difference of the vacuolar CoroNa Green intensity was observed in the second true leaf between the PNC treated cotton plants and the control group under salinity stress (200 mM NaCl, five days) (Fig. 6h). Moreover, under salinity stress (200 mM NaCl, five days), PNC treated cotton plants have significant higher leaf K^+^/Na^+^ ratio in both the first (9.16 ± 0.57 vs 1.91 ± 0.19) and the second (14.13 ± 1.79 vs 2.84 ± 0.17) true leaves than the NNP control group (Fig. 7c).

### PNC modulated the relative expression of genes related to Na^+^ and K^+^ transport

Under salinity stress (200 mM NaCl), no significant difference of the relative gene expression level of *SOS1* (salt overly sensitive 1, Na^+^/H^+^ antiporter for Na^+^ exclusion) and *NHX1* (Na^+^/H^+^ exchanger for vacuolar Na^+^ sequestration) was observed in cotton plant treated with and without PNC, in both the first and second true leaves (Fig. 7d). Whereas, PNC treated cotton plants showed significantly upregulated relative expression level of *HKT1* (high affinity K^+^ transporter for Na^+^ exclusion) than the control plants under salinity, either in the first or the second true leaf (Fig. 7d). In contrast to the upregulated *HKT1*, the relative expression level of *KOR* (K^+^ outward rectifying channel for K^+^ efflux) was significantly downregulated in PNC treated cotton plants compared with the control plants under salinity, either in the first or the second true leaf (Fig. 7d).

## Discussion

### Foliar application of nanoceria improves cotton salt tolerance

Our previous study tried nanoceria priming on cotton seeds and showed that compared with the control, nanoceria increased root length and biomass but not germination rate in cotton under salinity stress [26]. However, the experiments are conducted under paper roll condition and thus could not allow the build-up of normal cotton seedling plants. Here, hydroponic cultured cotton plants (two-leaf stage) were used to study the interactions between nanoceria and salinity stressed plants, which might give more clues about the mechanisms underline nanoceria improved plant salt tolerance. Also, in some semi-arid area, during the practice, good irrigation was usually operated at seed sowing stage but not last for seedling stage which is also critical for plant survival under hostile conditions. In this study, we showed that compared with the NNP control, nanoceria improved the phenotypic performance and increased biomass of cotton seedlings under salinity stress (Fig. 2A, B, Additional file 1: Figure S2). Also, the PNC treated cotton plants showed better chlorophyll content (Fig. 3) and photosynthetic performance (Fig. 4) than the NNP control under salinity stress. Overall, our results suggests that not only the seed nanopriming, but also foliar application of nanoceria at seedling plants could help to improve cotton salt tolerance. Moreover, our results added new information to support the possible addition of cotton to the suggest list of the potential crops for nano-enabled agriculture.

### Nanoceria improved ROS scavenging ability confers the enhanced cotton salt tolerance

ROS plays dual role in plants [47, 48]. ROS accumulation is known as a secondary stress in plants under salinity. Over-accumulated ROS could damage protein, DNA, membrane, and other biomolecules in plants [47]. ROSs in salt stressed plants mainly refer to hydrogen peroxide (H_2_O_2_), superoxide anion (^⋅^O_2_^−^), hydroxyl radicals (OH^⋅^) and singlet oxygen (^1^O_2_) [48]. The latter two cannot be scavenged by any known antioxidant enzymes. Among these ROSs, hydroxyl radicals are the most destructive ones, and are generated from hydrogen peroxide which reacts with transition metals through the Fenton reaction [48, 49]. Maintaining ROS homeostasis is important for plant salt tolerance. Plants evolved enzymatic and nonenzymatic antioxidant system to maintain ROS homeostasis [50, 51]. However, once the production overs the scavenging, ROSs start over accumulation which in turn impose negative effect on plant performance under stress. Thus, nanoparticles or nanomaterials with ROS scavenging ability, especially for hydroxyl radicals, may have the potential to maintain ROS homeostasis in plants under stress and thus to improve plant stress tolerance.

Here, we found that besides significant lower leaf MDA content, PNC treated plants showed increased antioxidant enzyme activities and decreased ROS content than the control under salinity stress (Fig. 4, 5 and Additional file 1: Figure S6). These results are in accordance with previous studies [6, 7, 13, 28], showing that through scavenging of ROS, nanoceria are able to help plants to resist stress conditions, e.g. salinity. Our results further reinforced the conception of nano-enabled agriculture, demonstrating the potential use of nanoparticles such as nanoceria to enhance salinity stress tolerance in plants, at least in cotton. This plant nanobiotechnology approach provides an alternative option of addressing current salinity issue in field and exploring the semi-arid area for cropping to the policy makers and farmers, especially cotton growers. Moreover, it should be noted that to benefit the sustainable agriculture, before the use of nanomaterials in crop plants, conducting safety assessment such as the effects of nanomaterials in the non-target organisms is necessary.

### Better maintained leaf K^+^/Na^+^ ratio is important for PNC improved cotton salt tolerance

Plant’s ability to maintain both cytosolic K^+^ and Na^+^ homeostasis is an essential trait indicating its salinity stress tolerance [15]. Massive over-accumulation of Na^+^ in the cytosol not only leads to Na^+^ toxicity, but also causes K^+^ efflux from the cytosol to the apoplast. K^+^ is a co-factor which is required for the activation of more than 50 enzymes [52]. It also plays important roles in cytosolic pH homeostasis, protein synthesis and cell activities e.g. stomatal opening and closure [53–55]. Under normal condition, the optimal K^+^ concentration in plant cell cytosol is about 100 mM [56, 57]. Plants can lose more than 50% K^+^ under salinity stress. Our previous work showed that nanoceria modulated the activity of ROS-activated NSCC channels to enable better mesophyll K^+^ retention and thus plant salt tolerance [13].

To date, the reported mechanisms for nanomaterials improved plant salt tolerance includes (1) maintaining ROS homeostasis by scavenging over-accumulated ROS or stimulating the activities of antioxidant enzymes [8, 28], (2) shortening the root apoplast barrier to allow more Na^+^ been translocated from root to shoot [6, 7], (3) modulating the activities of ion channels (nonselective cation channels, NSCC; and K^+^ outward rectifying channels, KOR) to enable better mesophyll K^+^ retention [13], 4) promoting the production of gas signaling molecules, such as nitric oxide [58]. As mentioned above, leaf cytosolic K^+^/Na^+^ ratio is a hallmark for plant salt tolerance [15]. However, the ability of cerium oxide nanoparticles to maintain cytosolic K^+^/Na^+^ ratio in salt stressed plant, especially the cash crop cotton, is less addressed. To maintain the leaf cytosolic K^+^/Na^+^ ratio, the ability to coordinate the level of K^+^ and Na^+^ in the cytosol is important. Here, we found that PNC treated plants showed better ability to maintain cytosolic K^+^ and total leaf K^+^ than the NNP control plants under salinity, either in the first or second true leaf (Figs. 6a–c, 7a). Also, *KOR* expression, a gene responsible for salt-induced K^+^ leak, is significantly downregulated in PNC treated plants than the NNP control under salinity stress (Fig. 7d), further confirming that PNC helped plants to maintain higher mesophyll K^+^ retention ability. This is in accordance with our previous study [13]. Also, the cytosolic Na^+^ level and total leaf Na^+^ in both the first and second true leaves of PNC treated plants is significantly lower than in NNP control plants under salinity stress (Figs. 6d–f and 7b). These results confirmed that PNC helped cotton plants to maintain a better leaf cytosolic K^+^/Na^+^ ratio and thus better performance under salinity stress. Overall, this work suggests that the ability to maintain the mesophyll cytosolic K^+^/Na^+^ ratio is a component of the mechanisms behind PNC enabled better plant salt tolerance. It adds more knowledge to our understanding of the mechanisms behind nanoceria-plant interactions regarding nano-enabled plant stress tolerance.

### PNC enabled lower mesophyll cytosolic Na^+^ is not associated with leaf vacuolar Na^+^ sequestration

Plants have different mechanisms to modulate cytosolic Na^+^ level, either by sequestrating Na^+^ into the vacuole [59] or excluding Na^+^ into the roots [20]. Here, our results showed that no increase, even decrease in the first true leaf, of vacuolar Na^+^ level in PNC treated plants than the NNP control under salinity stress (Fig. 6d–f). It suggests that at least in cotton, PNC enabled lower cytosolic Na^+^ is likely not enabled by possible high efficiency of vacuolar Na^+^ sequestration. Also, qPCR results showed that no difference in *NHX1* expression was found between PNC treated cotton plants and the NNP control under salinity (Fig. 7d). In contrast to the *NHX1*, an upregulation of *HKT1* was found in PNC treated cotton plants than the NNP control under salinity stress, suggesting that PNC enabled better leaf Na^+^ exclusion. This is in accordance with the results showing significantly lower leaf Na^+^ in PNC treated cotton plants than the NNP control under salinity stress (Fig. 7b). Overall, our results showed that in cotton plants, PNC enhanced leaf Na^+^ exclusion but not the ability of vacuolar Na^+^ sequestration under salinity stress. This is different to the mechanisms of PNC enabled salt tolerance in canola plants, by which shortening the apoplastic barrier to allow more Na^+^ to be transported into the shoot has been reported [7]. It suggests the complexity of the mechanisms behind PNC improved plant salt tolerance. It further calls the research attention on the different employed mechanisms of nanoparticle-plant interactions in different plant species. Moreover, besides shoot Na^+^ exclusion and vacuolar Na^+^ sequestration, limiting Na^+^ uptake from the root and its transport from root to shoot are also important mechanisms for plant salt tolerance [19, 20, 60]. Future studies are encouraged to study the contribution of Na^+^ exclusion from root uptake and xylem Na^+^ loading and unloading in nanomaterials improved plant salt tolerance.

### The different biological responses of leaves to PNC: more to be studied

One largely overlooked issue in nanoparticle-plant interactions is the possible different biological responses of leaves to the nanoparticles. In this work, we investigated the biological responses of the first and second true leaves of cotton plants to PNC under salinity stress. Our results showed that the biological responses of the first and second true leaves to PNC are different on some of the measured parameters in this study. For example, under salinity stress, the second true leaf have higher SOD and CAT activities than the first true leaf treated with PNC (Additional file 1: Figure S6a and S6b). Also, the second true leaf treated with PNC showed a significantly higher leaf K^+^/Na^+^ ratio than the first true leaf under salinity stress (Fig. 7c). Compared with the first true leaf, the amount of reduced cytosolic Na^+^ in leaves with and without PNC is 2.8 times higher in the second true leaf under salinity stress (Fig. 6g, h). Overall, it suggests that PNC may be able to protect the younger tissues, for example, the second true leaf in this study. These different biological responses between PNC treated first and second true leaves under salinity stress calls for more attention to study the nanoparticle-plant interactions at the level of tissue or organ level.

## Conclusions

The present work adds new knowledge about mechanisms behind nanoceria improved cotton salt tolerance at seedling stage. In the present study, our results showed that compared with the control group, PNC could scavenge ROS and enhance antioxidant enzyme activity in the first and second true leaves to maintain better ROS homeostasis in cotton plants under salinity stress. Besides maintaining ROS homeostasis, PNC helped salt stressed cotton plants to retain leaf K^+^ and to exclude over-accumulated leaf Na^+^, thus allowing the higher leaf K^+^/Na^+^ ratio in PNC treated plants than the NNP control plants under salinity stress. Combining the results from confocal imaging and qPCR experiments, our results suggest that not leaf vacuolar Na^+^ sequestration, but the enhanced leaf K^+^ retention and leaf Na^+^ exclusion are the main mechanisms behind PNC improved cotton salt tolerance. It also adds more knowledge about the mechanisms behind plant-nanoceria interaction.

## Supplementary Information

**Additional file 1: Table S1.** The used primers for the investigated genes in this study. **Figure S1.** The absorbance spectra and colour of PNC and DiI-PNC. a, the absorbance spectrum of the synthesized PNC and DiI-PNC. b and c, the colour of the synthesized PNC (b) and DiI-PNC (c).**Figure S2.** Confocal imaging of DiI fluorescence signals in Silwet-L77 treated cotton leaves. No DiI fluorescence signal was detected in cotton leaves treated with the surfactant Silwet-L77. **Figure S3.** The performance of salt stressed (200 mM NaCl, 5 days) cotton plants with and without PNC treatment. a and b, phenotypic performance of salt stressed cotton plants without (a) and with (b) PNC treatment. c and d, the plant height (c), and leaf dry weight (d) of the first and second true leaves of salt stressed cotton plants with and without PNC treatment. E, the whole plant fresh and dry weight of salt stressed cotton plants with and without PNC treatment. Mean ± SE (n = 12). Different lower-case letters indicate the significance level at 0.05. * means *P* < 0.05. **Figure S4.** The chlorophyll content of salt stressed (200 mM NaCl, 5 days) cotton plants with and without PNC treatment. a and b, chlorophyll a (a) and chlorophyll b (b) content in the first and second true leaves of salt stressed cotton plants with and without PNC treatment. Mean ± SE (n = 12). Different lower-case letters indicate the significance level at 0.05. **Figure S5.** The compatibility of PNC in cotton plants under non-saline condition. a and b, the phenotypic performance of cotton plants with and without PNC treatment, under non-saline condition after 28 days. c, CCI (chlorophyll content index) readout of the first and second true leaves of cotton plants with and without PNC treatment, under non-saline condition after 28 days. Mean ± SE (n = 8). NS means no significant difference. **Figure S6.** The antioxidant enzyme activities in salt stressed (200 mM NaCl, 5 days) cotton plants with and without PNC treatment. a-c, superoxide dismutase activities (a), catalase activities (b), and peroxidase activities (c) of the first and second true leaves of salt stressed cotton plants with and without PNC treatment. Mean ± SE (n = 12). Different lower-case letters indicate the significance level at 0.05.

## References

[1] Panta S, Flowers T, Lane P, Doyle R, Haros G, Shabala S (2014). Halophyte agriculture: success stories. Environ Exp Bot.

[2] Abdul R, Arfan A, Bin Safdar L, Mubashar Zafar M, Yang R, Amir S, Abbad S, Muhammad A, Wankui G, Youlu Y (2020). Salt stress induces physiochemical alterations in rice grain composition and quality. J Food Sci.

[3] Lima REM, Farias LFDL, Ferreira JFS, Suarez DL, Bezerra MA (2020). Translocation of photoassimilates in melon vines and fruits under salinity using C-13 isotope. Sci Hortic.

[4] Wang D, Lu X, Chen X, Wang S, Wang J, Guo L, Yin Z, Chen Q, Ye W (2020). Temporal salt stress-induced transcriptome alterations and regulatory mechanisms revealed by PacBio long-reads RNA sequencing in *Gossypium hirsutum*. BMC Genom.

[5] Karami A, Sepehri A (2018). Beneficial role of MWCNTs and SNP on growth, physiological and photosynthesis performance of barley under NaCl stress. J Soil Sci Plant Nutr.

[6] Rossi L, Zhang WL, Lombardini L, Ma XM (2016). The impact of cerium oxide nanoparticles on the salt stress responses of *Brassica napus* L.. Environ Pollut.

[7] Rossi L, Zhang WL, Ma XM (2017). Cerium oxide nanoparticles alter the salt stress tolerance of *Brassica napus* L. by modifying the formation of root apoplastic barriers. Environ Pollut.

[8] Zhao L, Lu L, Wang A, Zhang H, Huang M, Wu H, Xing B, Wang Z, Ji R (2020). Nano-biotechnology in agriculture: use of nanomaterials to promote plant growth and stress tolerance. J Agric Food Chem.

[9] Mahmoud AWM, Abdeldaym EA, Abdelaziz SM, El-Sawy MBI, Mottaleb SA (2020). Synergetic effects of zinc, boron, silicon, and zeolite nanoparticles on confer tolerance in potato plants subjected to salinity. Agronomy-Basel.

[10] Almutairi ZM (2016). Influence of silver nano-particles on the salt resistance of tomato (*Solanum lycopersicum*) during germination. Int J Agric Biol.

[11] Almutairi ZM (2016). Effect of nano-silicon application on the expression of salt tolerance genes in germinating tomato (*Solanum lycopersicum* L.) seedlings under salt stress. Plant Omics..

[12] Martinez-Ballesta MC, Zapata L, Chalbi N, Carvajal M (2016). Multiwalled carbon nanotubes enter broccoli cells enhancing growth and water uptake of plants exposed to salinity. J Nanobiotechnol.

[13] Wu H, Shabala L, Shabala S, Giraldo JP (2018). Hydroxyl radical scavenging by cerium oxide nanoparticles improves Arabidopsis salinity tolerance by enhancing leaf mesophyll potassium retention. Environ Sci Nano.

[14] Taha R, Mills D, Heimer Y, Tal M (2000). The relation between low K^+^/Na^+^ ratio and salt-tolerance in the wild tomato species *Lycopersicon pennellii*. J Plant Physiol.

[15] Almeida DM, Oliveira MM, Saibo NJM (2017). Regulation of Na^+^ and K^+^ homeostasis in plants towards improved salt stress tolerance in crop plants. Genet Mol Biol.

[16] Assaha DVM, Ueda A, Saneoka H, Al-Yahyai R, Yaish MW (2017). The role of Na^+^ and K^+^ tansporters in salt stress adaptation in glycophytes. Front Physiol.

[17] Wu HH, Shabala L, Zhou MX, Su NN, Wu Q, Ul-Haq T, Zhu J, Mancuso S, Azzarello E, Shabala S (2019). Root vacuolar Na^+^ sequestration but not exclusion from uptake correlates with barley salt tolerance. Plant J.

[18] Maathuis FJM (2014). Sodium in plants: perception, signaling, and regulation of sodium fluxes. J Exp Bot.

[19] Wu HH (2018). Plant salt tolerance and Na^+^ sensing and transport. Crop J.

[20] Munns R, Tester M (2008). Mechanisms of salinity tolerance. Annu Rev Plant Biol.

[21] United States Department of Agriculture ERS. Cotton Sector at a Glance; 2020.

[22] Li XW, Jin MG, Zhou NQ, Huang JO, Jiang SM, Telesphore H (2016). Evaluation of evapotranspiration and deep percolation under mulched drip irrigation in an oasis of Tarim basin, China. J Hydrol.

[23] Zhang DM, Li WJ, Xin CS, Tang W, Eneji AE, Dong HZ (2012). Lint yield and nitrogen use efficiency of field-grown cotton vary with soil salinity and nitrogen application rate. Field Crops Res.

[24] Peng J, Zhang L, Liu JR, Luo JY, Zhao XH, Dong HL, Ma Y, Sui N, Zhou ZG, Meng YL (2016). Effects of soil salinity on sucrose metabolism in cotton fiber. PLoS ONE.

[25] Razzouk S, Whittington WJ (1991). Effects of salinity on cotton yield and quality. Field Crops Res.

[26] An J, Hu P, Li F, Wu H, Shen Y, White JC, Tian X, Li Z, Giraldo JP (2020). Emerging investigator series. Molecular mechanisms of plant salinity stress tolerance improvement by seed priming with cerium oxide nanoparticles. Environ Sci Nano.

[27] Wu XW, Zhang Y, Lu YC, Pang S, Yang K, Tian ZM, Pei YX, Qu YQ, Wang F, Pei ZC (2017). Synergistic and targeted drug delivery based on nano-CeO_2_ capped with galactose functionalized pillar [5]arene *via* host-guest interactions. J Mater Chem B.

[28] Wu HH, Tito N, Giraldo JP (2017). Anionic cerium oxide nanoparticles protect plant photosynthesis from abiotic stress by scavenging reactive oxygen species. ACS Nano.

[29] Mitra RN, Gao RJ, Zheng M, Wu MJ, Voinov MA, Smirnov AI, Smirnova TI, Wang K, Chavala S, Han ZC (2017). Glycol chitosan engineered autoregenerative antioxidant significantly attenuates pathological damages in models of age-related macular degeneration. ACS Nano.

[30] Asati A, Santra S, Kaittanis C, Nath S, Perez JM (2009). Oxidase-like activity of polymer-coated cerium oxide nanoparticles. Angew Chem Int Ed Engl.

[31] Asati A, Santra S, Kaittanis C, Perez JM (2010). Surface-charge-dependent cell localization and cytotoxicity of cerium oxide nanoparticles. ACS Nano.

[32] Djanaguiraman M, Nair R, Giraldo JP, Prasad PVV (2018). Cerium oxide nanoparticles decrease drought-induced oxidative damage in sorghum leading to higher photosynthesis and grain yield. ACS Omega.

[33] Newkirk GM, Wu H, Santana I, Giraldo JP (2018). Catalytic scavenging of plant reactive oxygen species in vivo by anionic cerium oxide nanoparticles. J Vis Exp.

[34] Hu P, An J, Faulkner MM, Wu H, Li Z, Tian X, Giraldo JP (2020). Nanoparticle charge and size control foliar delivery efficiency to plant cells and organelles. ACS Nano.

[35] Wu HH, Santana I, Danise J, Giraldo JP (2017). In vivo delivery of nanoparticles into plant leaves. Curr Protoc Chem Biol.

[36] Safi M, Sarrouj H, Sandre O, Mignet N, Berret JF (2010). Interactions between sub-10-nm iron and cerium oxide nanoparticles and 3t3 fibroblasts: the role of the coating and aggregation state. Nanotechnology.

[37] Wu HH, Shabala L, Azzarello E, Huang YQ, Pandolfi C, Su N, Wu Q, Cai SG, Bazihizina N, Wang L (2018). Na^+^ extrusion from the cytosol and tissue-specific Na^+^ sequestration in roots confer differential salt stress tolerance between durum and bread wheat. J Exp Bot.

[38] Perez-Harguindeguy N, Diaz S, Garnier E, Lavorel S, Poorter H, Jaureguiberry P, Bret-Harte MS, Cornwell WK, Craine JM, Gurvich DE (2013). New handbook for standardised measurement of plant functional traits worldwide. Aust J Bot.

[39] Ferguson IB, Watkins CB, Harman JE (1983). Inhibition by calcium of senescence of detached cucumber cotyledons - effect on ethylene and hydroperoxide production. Plant Physiol.

[40] Yang J, Cao Y, Zhang N (2020). Spectrophotometric method for superoxide anion radical detection in a visible light (400–780 nm) system. Spectrochim Acta A Mol Biomol Spectrosc.

[41] Kashyap SP, Kumari N, Mishra P, Moharana DP, Aamir M, Singh B, Prasanna HC (2020). Transcriptional regulation-mediating ROS homeostasis and physio-biochemical changes in wild tomato (*Solanum chilense*) and cultivated tomato (*Solanum lycopersicum*) under high salinity. Saudi J Biol Sci.

[42] Chakraborty K, Singh AL, Kalariya KA, Goswami N, Zala PV (2015). Physiological responses of peanut (*Arachis hypogaea* L.) cultivars to water deficit stress: status of oxidative stress and antioxidant enzyme activities. Acta Bot Croat.

[43] Skowron E, Trojak M (2021). Effect of exogenously-applied abscisic acid, putrescine and hydrogen peroxide on drought tolerance of barley. Biologia.

[44] Peng Z, He SP, Sun JL, Pan Z, Gong WF, Lu YL, Du XM (2016). Na^+^ compartmentalization related to salinity stress tolerance in upland cotton (*Gossypium hirsutum*) seedlings. Sci Rep.

[45] Qin T, Liu SM, Zhang ZN, Sun LQ, He X, Lindsey K, Zhu LF, Zhang XL (2019). GhCyP3 improves the resistance of cotton to Verticillium dahliae by inhibiting the E_3_ ubiquitin ligase activity of GhPUB17. Plant Mol Biol.

[46] Chen XG, Lu XK, Shu N, Wang DL, Wang S, Wang JJ, Guo LX, Guo XN, Fan WL, Lin ZX, Ye WW (2017). *GhSOS1*, a plasma membrane Na^+^/H^+^ antiporter gene from upland cotton, enhances salt tolerance in transgenic *Arabidopsis thaliana*. PLoS ONE.

[47] Zhu JK (2016). Abiotic stress signaling and responses in plants. Cell.

[48] Mittler R (2017). ROS are good. Trends Plant Sci.

[49] Das K, Roychoudhury A (2014). Reactive oxygen species (ROS) and response of antioxidants as ROS-scavengers during environmental stress in plants. Front Environ Sci.

[50] Anjum SA, Tanveer M, Hussain S, Bao M, Wang LC, Khan I, Ullah E, Tung SA, Samad RA, Shahzad B (2015). Cadmium toxicity in Maize (*Zea mays* L.): consequences on antioxidative systems, reactive oxygen species and cadmium accumulation. Environ Sci Pollut Res.

[51] Mostofa MG, Hossain MA, Fujita M (2015). Trehalose pretreatment induces salt tolerance in rice (*Oryza sativa* L.) seedlings. Oxidative damage and co-induction of antioxidant defense and glyoxalase systems. Protoplasma.

[52] Wu HH, Zhang XC, Giraldo JP, Shabala S (2018). It is not all about sodium: revealing tissue specificity and signaling roles of potassium in plant responses to salt stress. Plant Soil.

[53] Gierth M, Maser P (2007). Potassium transporters in plants—involvement in K^+^ acquisition, redistribution and homeostasis. FEBS Lett.

[54] Wang Y, Wu WH (2010). Plant sensing and signaling in response to K^+^-deficiency. Mol Plant.

[55] Dreyer I, Uozumi N (2011). Potassium channels in plant cells. FEBS J.

[56] Leigh RA, Jones RGW (1984). A hypothesis relating critical potassium concentrations for growth to the distribution and functions of this ion in the plant-cell. N Phytol.

[57] Britto DT, Kronzucker HJ (2008). Cellular mechanisms of potassium transport in plants. Physiol Plant.

[58] Zhao G, Zhao Y, Luo W, Su J, Wei S, Yang X, Wang R, Guan R, Pu H, Shen W (2019). Nitrate reductase-dependent nitric oxide is crucial for multi-walled carbon nanotube-induced plant tolerance against salinity. Nanoscale.

[59] Apse MP, Aharon GS, Snedden WA, Blumwald E (1999). Salt tolerance conferred by overexpression of a vacuolar Na^+^/H^+^ antiport in Arabidopsis. Science.

[60] Tester M, Davenport R (2003). Na^+^ tolerance and Na^+^ transport in higher plants. Ann Bot.

